# Editorial: Advances in Cancer Stem Cell Biology

**DOI:** 10.3389/fgene.2021.655187

**Published:** 2021-03-26

**Authors:** Deema Hussein, Isabel M. Pires, Petra Krause, Hans-Juergen Schulten

**Affiliations:** ^1^Department of Medical Laboratory Technology, Faculty of Applied Medical Sciences, King Fahad Center for Medical Research, King Abdulaziz University, Jeddah, Saudi Arabia; ^2^Department of Biomedical Sciences, Faculty of Health Sciences, University of Hull, Hull, United Kingdom; ^3^Department of General, Visceral and Paediatric Surgery, University Medical Center, Goettingen, Germany; ^4^Center of Excellence in Genomic Medicine Research, Department of Medical Laboratory Technology, Faculty of Applied Medical Sciences, King Abdulaziz University, Jeddah, Saudi Arabia

**Keywords:** cancer, cancer stem cells, biomarkers, drug resistance, bioinformatics

The World Health Organization (WHO) reports that an estimated 9.6 million people died from cancer in 2018 (Bray et al., 2018). This estimate includes patients who had a diverse range of different types of cancers, including those arising in the lung, large intestine, stomach, liver, and breast cancer. For all of these tumors, the standard treatment options are surgery, radiotherapy, and chemotherapy. Several factors may influence the prognosis of a cancer patient. One particular factor that correlates with patients' survival is related to the biology of the tumor mass, i.e., whether the tumor grows slowly, fast, or has the capacity to relocate (Zhang et al., 2020; Thurmaier et al., 2021). The biologies of the different types of cancers are at the core connected through the abnormalities of 10 cellular pathways known as the hallmarks of cancer (Hanahan and Weinberg, 2011). Deregulation in these pathways is correlated with chemo and radio resistance (Buckley et al., 2020). A particular pathway that involves sustaining proliferative signaling and enabling cancer cells to behave similarly to embryonic stem cells has been of great interest in the area of translation oncology. Single-cell analysis of different cancers has shown clearly the existence and the diversity of a stem cell program in many tumor cells (Patel et al., 2014; Filbin et al., 2018). Targeting the diverse types of Cancer Stem Cells (CSCs) in *IDH*-wildtype Glioblastoma Multiforme (GBM) using combination therapy has been shown to be synergistic (Wang et al., 2019). Thus, characterizing the properties of CSCs is critical to improving future CSCs-targeting therapies. However, whether the activation of a deregulated stem cell program in CSCs is transient or stable remains to be addressed (Neftel et al., 2019).

The Advances in Cancer Stem Cell Biology topic aimed to provide a recent overview on the molecular biology of CSCs. Different approaches were used in published manuscripts from theory to bioinformatics and to experiments.

Using bioinformatics tools, Sang et al. aimed at identifying markers for CSCs that correlate with immune infiltrates in hepatocellular carcinoma (HCC) and poor patient survival. They utilized the Oncomine database, Gene Expression Profiling Interactive Analysis (GEPIA), and Integrative Molecular Database of Hepatocellular Carcinoma (HCCDB) to analyze the expression of hepatocellular CSC (HCSC) markers in 364 liver cancer samples. The correlation of HCSC markers to tumor-infiltrating immune cells was tested by Tumor Immune Estimation Resource (TIMER). Out of 10 differentially deregulated HCSC markers, 3 (*CD24, SOX9*, and *SOX12*) were highly expressed and had a positive correlation with poor prognosis. In contrast, the expression of *CD13, CD34*, and *ALDH1A1* was associated with prolonged overall survival. The authors noted that *SOX12* in particular might constitute a therapeutic target for hepatocellular carcinoma. Complementary to that work is the Li and Zhu manuscript, which reviewed recent advances in experimental studies on liver CSCs. They showed an update on the latest advances in experimental studies on non-coding RNAs (ncRNAs), oncogenes, and oncoproteins, with a particular focus on three pathways: the Wnt/β-catenin signaling pathway, phosphatidylinositol 3-kinase (PI3K)/protein kinase B (Akt) signaling pathway, and interleukin 6/Janus kinase 2/signal transducer and activator of transcription 3 (IL6/JAK2/STAT3) signaling pathway. Known associated roles for more than 30 CSC-related genes were discussed in detail. In particular, they conclude that octamer 4 (*OCT4*) and *NANOG* are important functional genes that play a pivotal role in liver CSC regulation and HCC prognosis.

Another bioinformatics-based paper, published by Tian et al., applied a weighted gene co-expression network analysis on gene expression data sets from head and neck squamous cell carcinomas (HNSCC) to define an mRNA expression-based stemness index consisting of genes that served as prognostic markers. Raw data for 643 samples were downloaded from the Cancer Genome Atlas (TCGA) database and the Gene Expression Omnibus (GEO) website. The study showed that the combined deregulated expression of eight stem-cell-related markers (*RGS16, LYVE1, hnRNPC, ANP32A, A1MP1, ZNF66, PIK3R3*, and *MAP2K7*) has a powerful capacity for overall survival prediction. They support their bioinformatics data by detecting the level of expression in HNSCC cell lines. The authors concluded that their proposed model could contribute to a better understanding of the role of HNSCC stem cells in developing targeted therapy.

An experimentally based approach was presented in the manuscript authored by Li et al. This work investigated the association of SET Domain Containing 2 (*SETD2*) gene mutations/variants with clinical features and prognosis in patients with Myelodysplastic syndrome (MDS). SETD2 is a transcriptional regulator and has been previously shown to be required for the self-renewal of hematopoietic stem cells (HSCs), and SETD2-deficient HSCs were shown to contribute to the development of MDS. Using targeted next-generation sequencing, the results indicated that out of 203 patients with MDS, 37 patients had *SETD2* gene mutations/variants, and these patients exhibited a significantly increased frequency of *TP53* mutations. Low expression of *SETD2* in patient tumor cells was identified as a risk factor for progression-free survival (PFS). The study concluded that *SETD2* deficiency contributes to genomic instability and is associated with unfavorable prognosis in patients with myelodysplastic syndrome.

Three more review articles were published in this collection. The first was a mini review by Azzarelli, which discussed the emerging 3D models of glioblastoma that overcome certain limitations of monolayer cultures. The author concluded that glioblastoma brain organoids provide the opportunity to study CSC lineages and serve as tools to predict tumor progression and treatment response. In a second review, Xu et al. discussed the role of N6-methyladenosine (m6A) in the differentiation of CSCs. The authors highlighted that targeting m6A modification of CSCs constitutes a yet not fully explored option for drug treatment of cancer. The third review was presented by Alhabbab, and it described how CSCs employ various mechanisms to modulate the immune system response. The review outlined the recent knowledge for the interactions between CSCs' common markers, including CD133, CD90, EpCAM, CD44, ALDH, and EGFRVIII, and the immune system. Current information on CAR T cell genetic engineering and signaling, CAR T cells, and the barriers in using CAR T cells as immunotherapy to treat solid cancers in the context of targeting CSCs were detailed.

Finally, in a theory-based article, Manzo investigated the nature of tumor growth within a mathematical model, which assumes tumors encompass CSCs that behave similarly to para-embryonic stem cells and divide into a hierarchic sequence of CSCs and non-CSCs. Tabulating theoretical data using this model, the author identified defined mathematical relationships between CSCs and non-CSCs that were similar to experimental data. The model explains tumor progression in a modular way that recalls the propagation of tumor spheres *in vitro*. Furthermore, the author discussed similar features, including nature form, dimension, cell distribution, and layer compartmentation for avascular tumors, tumor spheres, and preimplantation blastocysts. The author concluded that the presented mathematical model provides further support for the para-embryonic nature of the cancer process.

The research on CSCs is ongoing, and several concepts still remain to be addressed or fully explained. For example, what combinations of markers define different types of CSCs, and how does the “combined markers identity tag” correlate with therapeutic prognosis? Perhaps next-generation single-cell sequencing in combination with multiplex protein array technology could shed more light on the characteristics of CSCs and CSC markers. A unified CSC-specific interactive database for the mutational signatures and genomic instability of CSCs is likely to improve cancer research. Some questions remain: How do CSCs contribute to metastasis, and what are the therapeutics that can be given to combat CSCs movement and colonization? How can the gene expression profile of CSCs be stabilized and prevented from shifting in response to the microenvironment? What are the clinically relevant CSCs models that provide highly efficient translational protocols that can be utilized in a clinical setting?

Taken together, the variety of the authors' topic contributions, either by focused reviews, theoretical considerations, or research articles, has shed light on current advances in CSC biology and support further approaches for integrative CSC research.

## Author Contributions

All authors listed have made a substantial, direct and intellectual contribution to the work, and approved it for publication.

## Conflict of Interest

The authors declare that the research was conducted in the absence of any commercial or financial relationships that could be construed as a potential conflict of interest.
